# Comparison of various risk scores for the prognosis of hemorrhagic upper gastrointestinal mucosal disorder

**DOI:** 10.1186/s12245-020-00293-x

**Published:** 2020-07-29

**Authors:** Shinya Kita, Yasuyuki Shirai, Tomoharu Yoshida, Kei Shiraishi, Ayako Nakamura, Michitaka Kawano, Yoshihiro Kinoshita, Tatsuya Noguchi, Syunsuke Ito

**Affiliations:** 1 Fujii Hospital, 323, Tomo town, Fukuyama city, Hiroshima Prefecture 720-0201 Japan; 2 Kokura Memorial Hospital, 3-2-1, Asano, Kokurakita Ward, Kitakyusyu City, Fukuoka Prefecture 802-8555 Japan; 3 Shiraishi Gastrointestinal Clinic, 3-10-6, Kimachi, Kokurakita Ward, Kitakyusyu City, Fukuoka Prefecture 803-0851 Japan; 4 Saitama Medical University International Medical Center, 1397-1, Yamane, Hidaka City, Saitama Prefecture 350-1298 Japan; 5 Yamaguchi University, 1677-1, Yoshida, Yamaguchi City, Yamaguchi Prefecture 753-8511 Japan

**Keywords:** AIMS65 score, Charlson Comorbidity Index, Prognosis, Upper gastrointestinal bleeding, Upper gastrointestinal mucosal disorder

## Abstract

**Background:**

Various risk scores have been proposed that are useful for the management of upper gastrointestinal bleeding (UGIB), which is an important disease in emergency medicine. Few studies have examined the usefulness of Charlson Comorbidity index (CCI) in this disease, which evaluates the patient’s general condition by scoring the patient’s underlying disease. There have been no studies investigating the efficacy of CCI compared to other risk scores in the management of UGIB requiring endoscopic hemostasis.

**Methods:**

In addition to the Glasgow-Blatchford score, AIMS65 score, and Rockall score, we investigated the efficacy of the outcome prediction obtained by the original CCI and the updated CCI, scored only with respect to the underlying disease. We also examined the cutoff value when using the risk score. This retrospective study included 265 patients with hemorrhagic upper gastrointestinal mucosal lesions who underwent emergency endoscopic hemostasis during a 6-year period between 2011 and 2016 in our hospital.

**Results:**

The updated CCI and AIMS65 score correlated with prognosis in multivariate analysis (*p* = 0.002 and *p* = 0.003, respectively). In clinical practice, the prognosis might be worse if both updated CCI and AIMS65 score were 3 point or more.

**Conclusion:**

In addition to the AIMS65 score, the updated CCI can be a useful tool for managing upper gastrointestinal mucosal disorder bleeding that requires endoscopic hemostasis.

## Introduction

Hemorrhagic upper gastrointestinal mucosal disorder is often encountered in routine practice and may be potentially fatal. This disease can also arise from *H. pylori* infection, aspirin, non-steroidal anti-inflammatory drugs, and many other reasons even during treatment for other conditions. Thus, physicians in general should always be aware of it. Various prophylactic and therapeutic drugs have been developed to improve the treatment outcomes of hemorrhagic upper gastrointestinal mucosal disorder. However, the rates of upper gastrointestinal hemorrhage cases are still high, such that they are now considered to be a global emergency [1–4].

The efficacy of several different risk scores has been reported with respect to the management of UGIB. However, a clear predictive score has yet to be established [5, 6].

There are reports that patient complications contribute to the mortality rate in UGIB cases [7]. The Charlson Comorbidity Index (CCI) was proposed in 1984 as a tool for objective assessment of patient complications [8]. Along with the transition of disease management and progress of treatment, Quan et al. re-evaluated the CCI in 2011 and proposed the updated CCI, which produced modified scores [9] (Table 1). However, there have been no studies investigating the efficacy of CCI in the management of UGIB requiring endoscopic hemostasis.

**Table 1:** Charlson Comorbidity Index

Variable	Hazard ratio	Updated weight	Original weight
Myocardial infarction	0.99	0	1
Congestive heart failure^1^	1.91	2	1
Peripheral vascular disease^2^	1.1	0	1
Cerebrovascular disease^3^	1.1	0	1
Dementia	2.39	2	1
Chronic pulmonary disease^4^	1.28	1	1
Rheumatologic disease^5^	1.3	1	1
Peptic ulcer disease	1.08	0	1
Mild liver disease^6^	1.94	2	1
Diabetes without chronic complications^7^	1.12	0	1
Diabetes with chronic complications^8^	1.22	1	2
Hemiplegia or paraplegia^9^	2.26	2	2
Renal disease^10^	1.43	1	2
Any malignancy, including leukemia, and lymphoma	2.28	2	2
Moderate or severe liver disease^11^	3.83	4	3
Metastatic solid tumor^12^	6.01	6	6
AIDS/HIV	3.69	4	6
Maximum comorbidity score		24	29

^1^Exertional dyspnea, nocturnal dyspnea, response to medication

^2^Intermittent claudication, after bypass surgery, thoracic aortic aneurysm ≥ 6 cm

^3^Transient ischemia attack, cerebrovascular disease without sequelae

^4^Dyspnea with light exertion

^5^Systemic lupus erythematosus, polymyositis, mixed connective tissue disease, polymyalgia rheumatica, rheumatoid arthritis

^6^Cirrhosis without portal hypertension, chronic hepatitis

^7^Except diet therapy only

^8^With diabetic retinopathy or diabetic nephropathy or diabetic neuropathy, hospitalization history for diabetic ketoacidosis or diabetic coma

^9^Include not to be due to cerebrovascular disease

^10^Cre ≧ 3 mg/dL, dialysis, after kidney transplant, uremia

^11^Cirrhosis with portal hypertension

^12^No metastasis for past 5 years

In this study, we compared various scores (including the original and updated CCI) which are frequently used for predicting the prognosis of hemorrhagic upper gastrointestinal mucosal disorder.

## Materials and methods

The risk scores examined in this study were the Glasgow-Blatchford Bleeding Score (GBS) [10], full Rockall score [11], and AIMS65 score [12]; all of which are frequently used with respect to gastrointestinal bleeding. We also examined the original and updated CCI. Updated CCI has not been evaluated globally in UGIB cases.

We conducted a retrospective assessment of 265 cases of upper gastrointestinal mucosal disorders at our hospital, in which hemostasis was achieved through emergency upper gastrointestinal endoscopy, from January 1, 2011, to December 31, 2016, using electronic medical records. For each case, we investigated the various items necessary to obtain the adopted risk score. This study has been approved by the research ethics committee of the Kokura Memorial Hospital.

For each case, the sex, age, vital signs (including mental status), symptoms (melena and syncope), laboratory test findings, diagnosis, endoscopic stigma or bleeding, and underlying disease were recorded. Following these, we scored the various risk scores.

For vital signs, the systolic blood pressure and pulse rate were measured immediately before endoscopic treatment. Systolic blood pressure was subdivided into 4 groups: ≤ 89, 90–99, 100–109, and ≥ 110 for application to the GBS, full Rockall score, and AIMS65 score. Pulse rate was classified into two groups to adapt to the GBS and full Rockall score: ≤ 99 and ≥ 100.

Laboratory results of the hemoglobin, blood urea nitrogen, albumin, and PT-INR levels were adopted based on data collected immediately before endoscopic hemostasis was performed.

With respect to the underlying diseases, we considered the presence of renal dysfunction, liver dysfunction, myocardial infarction, congestive heart failure, dementia, peripheral vascular disease, chronic pulmonary disease, rheumatologic disease, peptic ulcer disease, stroke, hemi-paraplegia, diabetes, metastatic solid tumors, and any malignancy including leukemia and lymphoma. Disease severity was classified appropriately, as this was necessary for each score item. Renal function was classified into CKD G1-G2 (GFR ≥ 60), CKD G3a-G5 (eGFR < 60), and requiring hemodialysis or peritoneal dialysis, according to the kidney disease improving global outcomes (KDIGO) clinical practice guidelines. Liver dysfunction was defined as mild for cases up to Child-Pugh A and as moderate–severe for cases up to Child-Pugh B–C. Diabetes mellitus was classified based on the presence of complications (diabetic nephropathy, diabetic retinopathy, etc.).

Cases were classified into 2 groups: the survival group and the death group. The survival group included patients who were treated and did not require hospitalization or patients who could be discharged after hospitalization. The death group included patients who died even after being admitted to hospital, as well as patients who died from comorbidities other than those caused by UGIB.

### Statistical methods

Fisher’s exact test was used for comparisons between groups, and multivariate analysis was used for the forced input method for logistic regression analysis. It was evaluated using odds ratio (OR) and 95% confidence interval (CI), and significant differences were determined using *p* < 0.05.

Their correlation between prognosis and each score of AIMS65 and updated CCI were investigated. Each item included in each score was also examined for correlation with outcome. In addition, we analyzed the cutoff value for use in the outcome prediction for each score examined in this study, as it is important to grasp the exact cutoff value in clinical practice.

## Results

There were 265 cases of upper gastrointestinal mucosa disorders treated by hemostasis under emergency upper gastrointestinal endoscopy. 250 patients survived, while 15 died. Clinical characteristic and medication of each group is listed (Table 2). *H. pylori* infection is one of the important causes of gastrointestinal mucosal disorders. But it was difficult to make a clear distinction between past infection, current infection, and after eradication in retrospective research using electronic medical records. Therefore it is not listed. The risk score for each group is listed (Table 3). Regarding the cause of death, there was only one bleeding-related death. The majority causes of death were coexisting diseases with infections like pneumonia and septicemia or organ disorders such as heart failure (Table 4). The cause of death was decided by each clinician.

**Table 2:** Clinical characteristics of the patient in the present study

			Total	Survival	Death	*p* value
Characteristic	Sex (%)	Male/Female	195 (73)/70 (26.4)	185 (74)/65 (26)	10 (66.7)/5 (33.3)	0.551
	Age (mean ± SD)		71.2 ± 12.7	71.1 ± 12.7	73.3 ± 12.2	0.509
Drug	Aspirin (%)		118 (44.9)	111 (44.8)	7 (46.7)	0.131
	Aspirin + Clopidogrel (%)		45 (17.1)	44 (17.7)	1 (6.7)	0.479
	Anticoagulant drugs (%)		72 (27.4)	65 (26.2)	7 (46.7)	0.131
	Other antiplatelet drugs (%)		10 (3.8)	10 (4.0)	0 (0.0)	> 0.999
	NSAIDs (%)		53 (20.1)	50 (20.1)	3 (20.0)	> 0.999
	Preventive drugs (%)	H_2_RA/PPI/MPAs	31 (11.7)/51 (19.2)/21 (7.9)	30 (12.0)/46 (18.4)/19 (7.6)	1 (6.7)/5 (33.3)/2 (13.3)	0.047

*NSAIDs* non-steroidal anti-inflammatory drugs, *H2RA* histamine H2 receptor antagonist, *PPI* proton pomp inhibitor, *MPAs* mucosal protective agents

**Table 3:** Comparison of risk scores between survival and death groups

Risk score	Survival group	Death group	*p* value
AIMS65 score (mean ± SD)	1.4 ± 0.9	2.3 ± 1.0	0.0001
Glasgow-Blatchford score (mean ± SD)	10.5 ± 3.4	13.0 ± 3.1	0.005
Rockall score (mean ± SD)	6.4 ± 2.0	7.9 ± 2.8	0.005
Charlson Comorbidity Index (mean ± SD)	2.1 ± 1.8	3.9 ± 2.4	0.0002
Updated Charlson Comorbidity Index	1.1 ± 1.5	2.9 ± 2.1	0.00002

**Table 4:** List of mortalities after endoscopic hemostasis

Number	Comorbidities	Onset	Cause of death
1	HD, LD (mild), HF (acute), stroke	Inpatient	Aspiration pneumonitis
2	Metastatic solid tumor	Inpatient	Lung cancer
3	CKD	Inpatient	Septicemia
4	CKD	Inpatient	MODS
5	HD, HF (obsolete), stroke	Inpatient	Infectious endocarditis
6	CKD, MI (obsolete), DM (with chronic complication), stroke	Outpatient	Heart failure
7	MI (obsolete), HF (obsolete)	Inpatient	Asphyxia
8	LD (mild), DM (without chronic complication)	Outpatient	Hemorrhagic anemia
9	CKD, PVD, DM (with chronic complication)	Inpatient	Septicemia
10	HD, MI (obsolete), HF (obsolete), stroke, PVD, DM (without chronic complication)	Inpatient	DIC
11	CKD	Outpatient	Acute pneumonia
12	LD (severe), solid tumor	Outpatient	Acute pneumonia
13	CKD, MI (obsolete), HF (acute), PVD	Inpatient	MODS
14	CKD, PVD	Outpatient	MODS
15	HD, Liver disease (severe)	Outpatient	MODS

*HD* hemodialysis, *MI* myocardial infarction, *HF* heart failure, *CKD* chronic kidney disease (Grade 3a-Grade 5 with no dialysis), *DM* diabetes mellitus, *PVD* peripheral vascular disease, *LD* liver disease, *MODS* multiple organ dysfunction syndrome, *DIC* disseminated intravascular coagulation syndrome

### Consideration of the factors related to prognosis and correlation with risk scores

**Fig. 1:**
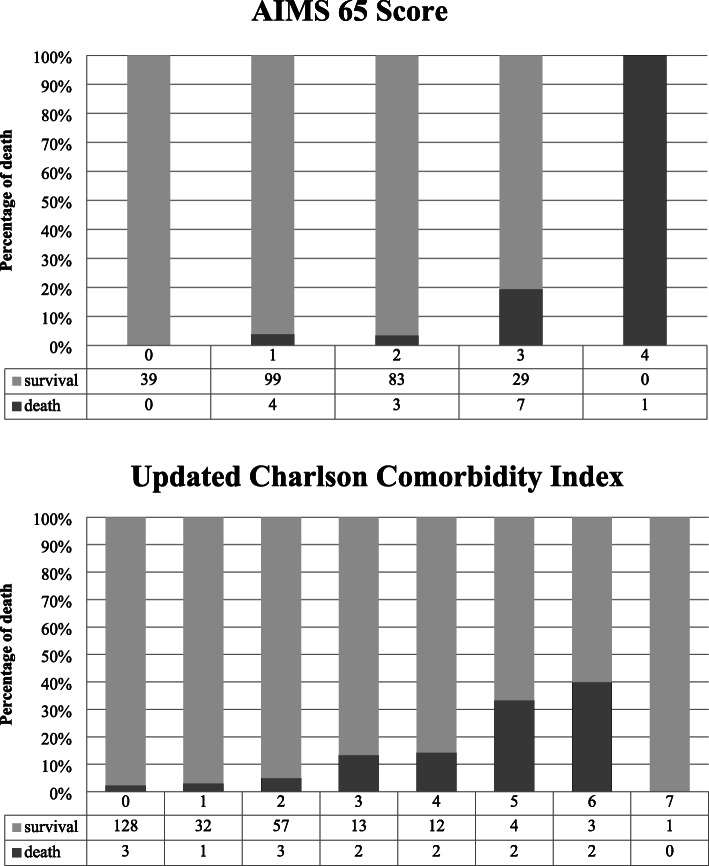
Outcomes for AIMS65 score and updated CCI

We compared the risk scores in each group using univariate analysis. The AIMS65 score (*p* = 0.0004, OR 3.327, 95% CI 1.702–6.503), GBS (*p* = 0.004, OR 1.347, 95% CI 1.099–1.651), and full Rockall score (*p* = 0.006, OR 1.395, 95% CI 1.098–1.773) correlated with the outcome. The original CCI (*p* = 0.001, OR 1.505, 95% CI 1.187–1.908) and updated CCI (*p* = 0.0002, OR 1.674, 95% CI 1.280–2.188) also exhibited a correlation with prognosis. Subsequently, we examined the relationship between these risk scores and prognosis using the forced input method for logistic regression analysis. We found that the updated CCI (*p* = 0.002, OR 1.586, 95% CI 1.177–2.136) and AIMS65 score (*p* = 0.003, OR 2.716, 95% CI 1.394–5.292) exhibited a strong correlation with outcome compared to other scores (Table 5). We show the outcomes of both AIMS65 score and updated CCI for each scoer (Fig. 1).

**Table 5:** Relationship between various scores and outcomes using univariate analysis and logistic regression analysis

Risk score	OR	95% Cl	*p* value
Univariate analysis			
Charlson Comorbidity Index (per 1)	1.505	1.187 , 1.908	0.001
Updated Charlson Comorbidity Index (per 1)	1.674	1.28 , 2.188	0.0002
Glasgow-Blatchford score (per 1)	1.347	1.099 , 1.651	0.004
Rockall score (per 1)	1.395	1.098 , 1.773	0.006
AIMS65 score (per 1)	3.327	1.702 , 6.503	0.0004
Logistic regression analysis			
Updated Charlson Comorbidity Index (per 1)	1.586	1.177 , 2.136	0.002
AIMS65 score (per 1)	2.716	1.394 , 5.292	0.003

We also investigated the cutoff value of these two risk scores. Both updated CCI and AIMS65 score showed poor outcome when the score was ≥ 2.5 points. In clinical practice, 3 points were assumed to be cutoff value (Table 6).

**Table 6:** Optimal thresholds in prediction of outcome

Score	Cutoff	Sensitivity (%)	Specificity (%)	PPV (%)	NPV (%)
Updated Charlson Comorbidity Index	≥ 2.5	53.3	86.8	19.5	96.9
AIMS65 score	≥ 2.5	53.3	88.4	21.6	96.9

In terms of the items required for various scores, we examined the relationship with prognosis using the forward selection method for logistic regression analysis and found that albumin level (*p* = 0.012, OR 0.331, 95% CI 0.140–0.787), peripheral vascular disease (*p* = 0.004, OR 8.649, 95% CI 1.955–38.254), and severe liver impairment (*p* = 0.001, OR 93.585, 95% CI 5.841–1499.327) were correlated with outcome (Table 7).

**Table 7:** Relationship between each items and outcomes using logistic regression analysis

Item	OR	95% Cl	*p* value
Albumin (per 1)	0.331	0.14 , 0.787	0.012
Liver function (Child B-C)	93.585	5.841 , 1499.327	0.001
Peripheral vascular disease	8.649	1.955 , 38.254	0.004

## Discussion

### Score development process and research results

In upper gastrointestinal hemorrhage cases, the necessity for hospitalization and endoscopic intervention, recurrence of bleeding, and outcome are essential pieces of information when considering treatment management. Based on these perspectives, various risk scores have been created, and their usefulness in clinical practice has been studied.

The Rockall score, incorporating clinical and endoscopic elements, was developed in 1996 to predict mortality in UGIB. In 2011, a newer and simpler acronymic risk score, AIMS65, was developed to predict mortality. To identify high-risk patients requiring immediate intervention and low-risk patients that can be safely discharged, the GBS was established in 1997.

To compare the predictive accuracy and clinical utility of these risk scores, an international multicenter prospective study was implemented. GBS was determined to be the most effective predictor for judging whether medical intervention, including endoscopic treatment, is appropriate. The Progetto Nazionale Emorragie Digestive (PNED) [13] and AIMS65 scores were reported to offer greater utility than the other scores with respect to prognostic prediction. Re-bleeding and hospitalization periods were also examined; however, the scores were not correlated [6]. We excluded PNED score from this study because retrospective research using electronic medical records could not determine the American Society of Anesthesiologists status [14] required in the score.

Although not frequently used for UGIB, the original CCI was proposed in 1984; it scores 17 items relating to a patient’s underlying disease and severity to evaluate the patient’s general condition objectively. In 2011, the updated CCI decreased items relating to underlying diseases and changed the score, with improving medical quality. The usefulness of the updated CCI in developed countries was reported in 2011 [9]. CCI is a score that is clinically applicable for all diseases; however, there are few reports discussing its utility with respect to UGIB cases. Furthermore, there have been no studies comparing CCI with the other risk scores in this field.

We previously reported that the original CCI was useful for predicting the prognosis of low-dose aspirin-induced bleeding gastroduodenal ulcers treated with endoscopic hemostasis at our hospital [15]. Based on the results of this previous study, we propose that the updated CCI may also be useful for predicting prognosis in upper gastrointestinal mucosal disorder patients. In fact, Wierachowski et al. reported that CCI was significantly higher in the death group compared to the survival group in case of gastrointestinal bleeding [16]. Therefore, we compared various risk scores, including the original CCI and the updated CCI in gastrointestinal mucosal disorder patients, to predict their prognosis.

In this study, the updated CCI and AIMS65 score showed significant correlation with prognosis using multivariate analysis. The updated CCI had greater correlation with prognosis than the original CCI because the scores were altered in consideration of advancements in medical technology. We examined whether updated CCI or AIMS65 score correlated more with prognosis by the Delong’s test using AUC value, but no significant difference (*p* = 0.969) was observed. In clinical application, both suggested worsening outcome, at 3 points or more.

### Why the AIMS65 score is useful in predicting prognosis

Among the items in AIMS65 score, the factor that contributed most to the outcome was the albumin level. Albumin is essential for maintaining health and has numerous important physiological functions. Hypoalbuminemia is an important prognostic factor for chronic disease patients [17]. The usefulness of the albumin level has also been verified in the context of the management of gastrointestinal bleeding patients. Gonzalez-Gonzalez et al. reported a correlation (*p* = 0.001, OR 5.230, 95% CI 2.099–13.029) with the prognosis of serum albumin values in elderly peptic ulcer patients; thus, the albumin level may be useful as a prognostic marker [18].

### Why CCI is useful in predicting prognosis

Severe liver dysfunction and peripheral vascular disease, items which are included in the score, were strongly correlated with prognosis. The reason why original CCI had better correlation with prognosis than the other frequently used risk scores except AIMS65 was because both the above two items were included in the score. There are many reports on liver dysfunction’s correlation with prognosis in UGIB; however, few have reported on the correlation of peripheral vascular disease with prognosis. The updated CCI score decreases the weight of peripheral vascular disease to 0 and increases the weight of severe liver disease to 4 from 3. The reason why updated CCI also correlated with prognosis even excluding peripheral vascular disease was that the score for severe liver disease was increased and combined effects of various score changes taking into account medical progress, a single item did not correlate with prognosis in this study.

In addition, there are two more reasons why CCI correlated with prognosis in this study: it is a score that evaluates the number of complications, as well as the severity of complications. Regarding number of co-morbidities, Krag et al. investigated the correlation between risk factors and frequency of gastrointestinal bleeding in seriously ill patients and found that the risk of bleeding was high in three or more severe cases with co-morbidities (OR 5.2, range 2.7–22.8) [19]. Regarding the severity of co-morbidities, the CCI changes score with the severity of liver disease. Juan et al. reported that UGIB mortality increased as liver function worsen [20]. This study also demonstrated that severe liver dysfunction had an impact on prognosis.

### Limitation

We were unable to obtain effective detection power due to the small sample size with respect to the number of examined items. It is thus necessary to improve the detectability with further accumulation of cases. In addition, because this was a retrospective study, there was no clearly established treatment protocol. As such, the possibility that treatment decisions may have affected prognosis cannot be eliminated as a confounding factor. Furthermore, we gathered data at a single center, so this study was not universal. In the future, studies producing high-level evidence through prospective assessment and multicenter collaboration will be necessary. Our hospital specializes in cardiovascular disease; as such, this study included more patients with severe peripheral vascular disease compared to other institutions. The background of these patient characteristics may have influenced the outcome and needs to be interpreted carefully. For this study, case selection was not performed at the stage of patient enrollment.

## Conclusion

This study reaffirmed the usefulness of the AIMS65 score, which was previously found to be useful for predicting the prognosis of UGIB. In addition, our results demonstrated the usefulness of the updated CCI. Both the updated CCI and AIMS65 are scores that take into account the patient’s general condition. Therefore, in case of hemorrhagic upper gastrointestinal mucosal disorder, systemic management that considers the patient condition, in addition to hemostasis management, is very important.

In conclusion, CCI, especially updated CCI, can be an effective risk score in predicting prognosis of hemorrhagic upper gastrointestinal mucosal disorder.

## Data Availability

Please contact author for data requests.

## Abbreviations

CCI: Charlson Comorbidity Index
UGIB: Upper gastrointestinal bleeding
GBS: Glasgow-Blatchford Bleeding Score
KDIGO: Kidney disease improving global outcomes
PNED: Progetto Nazionale Emorragie Digestive
HD: Hemodialysis
MI: Myocardial infarction
HF: Heart failure
CKD: Chronic kidney disease
DM: Diabetes mellitus
PVD: Peripheral vascular disease
LD: Liver disease
MODS: Multiple organ dysfunction syndrome
DIC: Disseminated intravascular coagulation syndrome
